# Omicron infection elicits a broad neutralizing response in hospitalized patients with COVID-19

**DOI:** 10.1172/JCI165034

**Published:** 2022-12-01

**Authors:** Elizabeth M. McNally

In this issue of the *JCI*, Linderman and colleagues evaluated neutralizing antibody responses in hospitalized patients with SARS-CoV-2 infections (1). Since early in the pandemic, it has been observed that COVID-19 infections resulting in hospitalization generate a stronger antibody response compared with infections in nonhospitalized individuals (based on community-acquired blood samples) (2). At the same time, the breadth of antibody responses differs across SARS-CoV-2 variants (3), with some viral subtypes eliciting antibodies with little cross-protection to other viral subtypes. It was for this reason that an Omicron subvariant is now present in the newest bivalent vaccines. The Linderman study examined samples from hospitalized patients collected in the period from the latter half of 2021 to early 2022, comparing responses to Delta and the Omicron subvariants BA.1 and BA.2. For the majority of the cohort, the authors determined the specific SARS-CoV-2 viral subtype by sequencing. Furthermore, the immune response was measured as true neutralizing response to live virus, as opposed to surrogate neutralization tests (1).

In unvaccinated study participants, Delta infection elicited an antibody response highly skewed toward the Delta subvariant, with distinctly lower neutralizing response to Omicron. For vaccinated participants, Delta infection resulted in antibodies toward the original WA1 strain and Delta, reflecting both vaccine-induced protection and response to the Delta infection. Notably, all Delta-infected participants had lower neutralizing titers to Omicron subvariants BA.1 and BA.2. In contrast, patients infected with Omicron generated a more balanced response, with neutralizing antibodies against Delta, BA.1, and BA.2 (1).

The total hospitalized cohort (*n* = 187) included unvaccinated individuals (43%). Of the vaccinated patients who required hospitalization, one-third were immunosuppressed. Two-thirds of the immunosuppressed, vaccinated patients had a history of solid organ transplantation or hematologic malignancy with immunosuppression, consistent with a high degree of immunocompromise (1).

In the US, widespread population immunity has developed due to a combination of both natural and vaccine-induced immune responses to COVID-19 (4). This population immunity has allowed US public health strategies to focus on hospital bed use as a major determinant of recommendations regarding travel, large gatherings, and masking. The work of Linderman stands out for its focus on hospitalized COVID-19 patients, since it is the number of hospital beds occupied by COVID-19 patients that can shift public health guidance for pandemic-related restrictions (1).

The SARS-CoV-2 Delta wave from summer 2021 through December 2021 provided ample evidence for infection after either prior COVID-19 infection (reinfection) or mRNA vaccination (vaccine breakthrough). Reinfections and breakthrough infections in COVID-19 are part of the known challenges of protecting against reinfection from coronaviruses (5). Vaccination and natural infection both provide substantial protection against serious reinfection, as defined by the need for hospitalization. The broader immune response to Omicron infection, with some crossover to Delta, as reported by Linderman et al., could partially derive from prior natural exposure. The authors raise the question as to whether a one-time immunization with an Omicron-containing vaccine will provide insufficient protection and whether reimmunization with Omicron will be needed (1). Another view maintains that vaccines expressing spike proteins from multiple SARS-CoV-2 subvariants could promote an immune response against the most common and protective regions of the SARS-CoV-2 spike protein, and therefore work better (6). An alternative idea posits that repeated exposure, both natural and vaccine induced, leads to a cumulative dose that creates a more durable immunity. Time will tell.

## Version 1 12/01/2022

Electronic publication
